# Draft Genome Sequence of a Metronidazole-Resistant Bacteroides fragilis Strain Isolated in Ecuador

**DOI:** 10.1128/MRA.01125-19

**Published:** 2019-12-05

**Authors:** Jeannete Zurita, Gabriela Sevillano, Ariane Paz y Miño, Francisco Flores, Marcela Bovera

**Affiliations:** a Facultad de Medicina, Pontificia Universidad Católica del Ecuador, Quito, Ecuador; b Unidad de Investigaciones en Biomedicina, Zurita & Zurita Laboratorios, Quito, Ecuador; c Departamento de Ciencias de la Vida y Agricultura, Universidad de las Fuerzas Armadas–ESPE, Sangolquí, Ecuador; d Centro de Investigación de Alimentos (CIAL), Facultad de Ciencias de la Ingeniería e Industrias, Universidad UTE, Quito, Ecuador; e Hospital de los Valles, Quito, Ecuador; Broad Institute

## Abstract

Here, we report the draft genome sequence of Bacteroides fragilis strain Z&Z143, a metronidazole-resistant bacterium isolated from a blood culture from an Ecuadorian patient hospitalized in a medical institution in Quito, Ecuador. We describe a new variant of the *nim* genes, which is associated with metronidazole resistance.

## ANNOUNCEMENT

Bacteroides fragilis is a predominant organism in the colon microbiota (1). It is one of the most frequently isolated species from clinical infections and bacteremia (2). The most common antimicrobial drug for the treatment of B. fragilis infections is metronidazole (3). Worldwide, the prevalence of metronidazole-resistant B. fragilis has been reported to be between 0.5% and 7.8% (4). The resistance is often conferred by the *nim* genes, which encode 5-nitroimidazole reductase (5). Before this analysis, 11 variants of the *nim* gene (*nimA* to *nimK*) had been identified, mainly in *Bacteroides* spp. and rarely in other anaerobes (6).

B. fragilis was isolated from a blood culture from a patient who had abdominal surgery and was hospitalized in Quito, Ecuador. It was obtained using a Bactec lytic anaerobic blood culture bottle (Becton, Dickinson, USA) after routine subculture on a BBL *Bacteroides* bile esculin (BBE) agar plate (Becton, Dickinson) at 37°C in an anaerobic atmosphere for 24 h. The API 20A system (bioMérieux, Marcy l’Étoile, France) was applied for the identification of B. fragilis (Z&Z143). MICs were determined using a Sensititre anaerobe MIC ANO2B plate (Thermo Fisher Scientific, USA). The results were interpreted according to Clinical and Laboratory Standards Institute (CLSI) guidelines (7). Total DNA from the B. fragilis Z&Z143 strain, harvested from BBE agar, was extracted using the High Pure PCR template preparation kit (Roche Diagnostics, Switzerland), according to the manufacturer’s instructions. The TruSeq Nano DNA library preparation kit (Illumina) was used to prepare libraries that were sequenced using the Illumina HiSeq system in 100-bp paired-end runs, producing a total of 10,317,898 reads. The Trimmomatic version 0.39 tool was used to trim and filter low-quality reads. Assembly was conducted with Velvet 1.2 (https://cge.cbs.dtu.dk/services/Assembler/) (8), resulting in 75 contigs >200 bp in size and an estimated genome size of 5 Mb. Antimicrobial resistance genes were determined using ResFinder (9). Default parameters were used for all software.

The isolated B. fragilis strain was characterized according to its phenotypic and genotyping resistance (Table 1).

**TABLE 1:** Phenotypic and genotypic antimicrobial susceptibility profile

Antimicrobial	MIC breakpoint (mg/liter)	Resistance interpretive category^*a*^	Resistance gene
Ampicillin-sulbactam	16/8	R	*cepA*
Amoxicillin-clavulanic acid	4/2	S	
Cefotetan Na	≤4	S	
Penicillin	≥4	R	
Imipenem	≤0.12	S	
Meropenem	≤0.5	S	
Clindamycin	≥8	R	*ermF*
Cefoxitin	4	S	
Metronidazole	≥16	R	*nimL*
Chloramphenicol	≥64	R	Chloramphenicol-acetyltransferase
Ampicillin	≥16	R	
Piperacillin	128	R	
Tetracycline	≥8	R	*tetQ*
Mezlocillin	128	NA	
Piperacillin-tazobactam	2/4	S	

a The results were interpreted using CLSI M100, 29th ed. (7). R, resistant; S, susceptible; NA, CLSI breakpoints were not available.

The draft genome of metronidazole-resistant B. fragilis Z&Z143 has a length of 5,158,698 bp, with an *N*_50_ value of 196,742 bp, genome coverage of 200.0×, and G+C content of 43.1%. A total of 4,320 protein-coding genes and 71 RNA-coding sequences were detected after manual inspection of the annotations using the NCBI Prokaryotic Genome Annotation Pipeline (PGAP) (https://www.ncbi.nlm.nih.gov/genome/annotation_prok/) (10).

Functional annotation with the PGAP highlighted genes that were associated with the nitroimidazole reductase protein, which is responsible for the metronidazole resistance in this strain. A BLAST search of the Z&Z143 nitroimidazole resistance gene resulted in a nucleic acid identity of 89% with the *nimC* gene of Bacteroides thetaiotaomicron (GenBank accession number NG_048013) and 83% with the *nimF* gene of Bacteroides vulgatus (GenBank accession number AJ515145). The presence of this *nim* gene in B. fragilis was further confirmed by PCR using the NIM-3 and NIM-5 primers (11). The new variant of the *nim* gene conferring metronidazole resistance described in this study was named *nimL* (GenBank accession number MK251988).

The increasing diversity of *nim* gene variants is of great concern because there is a possibility for new emerging multidrug-resistant *Bacteroides* strains. Furthermore, the activity of metronidazole, traditionally used as an empirical therapy for anaerobic infections, might be compromised.

### Data availability.

The genome sequence was deposited under GenBank accession number SRKB00000000, BioProject number PRJNA530267, SRA accession number SRR10193381, and BioSample accession number SAMN11310039.
